# Surgical Diseases of the Mouth

**Published:** 1871-08

**Authors:** 


### ARTICLE VII.

### *Surgical Diseases of the Mouth.*

*Glottis* is an inflammation of the tongue, being the result, in general, of mercurialization. The tongue becomes enormously swollen, enlarged from the infiltration of serum; the increase in size, in some cases, having been known to threaten death by suffocation. The accompanying symptoms are, profuse salivation, inability to swallow, or even speak, with the organ hanging out of the mouth, as in prolapsus. The difference is this, in glottis there is a comparatively sudden invasion, whilst the latter is gradual. Our remedies, particularly *Apis*, act like magic in these cases, and the harsh allopathic procedure of slitting the tongue is seldom if ever called for.

*Prolapsus of the Tongue.*—This is a condition in which the tongue hangs out of the mouth permanently. The patient has no power to retain it in the mouth any length of time. Prolapsus may be congenital, or acquired. The teeth, and even the jaws, are frequently pushed forward, the edges of the teeth meeting at an angle very similar to that noticed in the horse. Should this condition be allowed to remain unimproved, the jaw will be permanently distorted. There are cases of evident hypertrophy of the whole organ, but the fault usually seems to lie in a want of power in the retractor muscles. The tongue is of a dark or purplish color, dry, much swollen, and a constant dribbling of saliva. I am not certain that this is a disease—if such it may be called—that is curable by medicine alone, but think operative measures will have to be conjoined with the other treatment. Still, it being a case in which there is no great urgency, time enough may be taken to test the remedies. This is one of those diseases which are of so rare occurrence, and the results of which are so very unsatisfactory, partly from the fact of but few being willing to give time enough to properly test the matter, that we know but little about the effects of remedies. We have to reason by analogy, and such reasoning in medicine is far from being reliable. I am not cognizant of a single case having been reported where a *perfect cure followed the use of remedies.*

*Abscess of the Tongue.*—This is also a very rare disease, and is not easily diagnosed when seen. Like all other similar diseases, the pus must be evacuated at once. Erichsen speaks of a case in which "there was an elastic fluctuating tumor of slow growth, about the size of a small plum, situated deeply in the centre of the tongue." I never saw a case, but have been informed that as the tongue is not discolored is not at all easy to tell whether it be an abscess or a tumour from some other cause. Dr. Duncan had a case of abscess of the tongue. The abscess was situated in the centre of the tongue. It was about the size of a large pea and presented a red base; cause, cold; remedy, *Hepar.*

*Ranula* is a most interesting form of a tumor, found under the tongue, to one side of the frænum. There are two varieties, first, a cyst, containing fluid, and often as large as a walnut. The fluid is serous and of a glairy nature; the walls of the cyst are very thin, almost transparent, and small vessels can be distinctly seen ramifying on the surface. Many consider this a simple dilatation of Wharton's duct, but that is not the general belief. Some pathologists, among them Paget, think many of these ranulæ, "are probably formed by dilatation of the sub-maxillary duct, obstructed by calculi or otherwise; others by abnormal development of distinct cysts, or possibly of a bursœ between the muscles of the tongue." The second form is that of a true encysted tumor, the contents of more or less consistency, and walls firmly adherant to the adjacent parts.

*Tumors* of all varieties are found in this situation, but they do not need any special mention, but should be treated according to the indications.

*Fissures or cracks in the tongue* are found on the sides of the organ, and are usually dependent upon the irritation caused by the root of a molar tooth; at other times they are the product of dyspepsia, syphilis, &c. In cases where the general health is low, or the system is tainted with syphilis, struma, or scorbutis, these cracks are converted into large and foul ulcers, which extend rapidly, attended by offensive breath, and constant flow of offensive saliva; in fact they are true phagedœna. The surrounding parts of the organ are swollen and puffy, and of a dusky-red color. Death frequently results from the excessive discharge, and the inability to take food, though in many cases rupture of the artery is the cause.

*In Psoriasis* the tongue become shriveled and dry, much indurated, with the surface covered with cracks; with patches, also, on the surface, of a dirty-white color, irregular in size and shape, varying from an inch in diameter to the size of a pea. This condition is sometimes met as a manifestation of secondary syphilis, and certainly, to the eye, looks like a purely local disease as such a thing can be. Another syphilitic condition is one that has not been named, to my knowledge, and is described by Erichsen in the following manner: "A glazed and warty appearance, as if covered with a layer of boiled sago, the mucous membrane being œdematous, elevated, and papilated, yet at the same time glassy and semi-transparent, and without induration."

*Syphilitic tubercle* is an indurated and irregularly circumscribed tumor, situated deep in the substance of the tongue, usually near the centre of tip, and feels round to the touch. The integumentary covering of the tumor is of a coppery or dusky red color, surrounded by cracks, but it does not ulcerate, discharge, or destroy the organ.

*Cancer of the tongue* so closely resembles some of the syphilitic affections of this organ that great care must be exercised in forming a diagnosis. One thing worth remembering is, that the syphilitic tubercle differs from the cancerous in being situated deeply in the substance of the part, while the latter is more superficial and is found about the edges and tip. (See Ulcers.)

*Ulcers.*—There are the phagedœnic, the syphilitic, and the cancerous, and may be distinguished by attention to the following signs: *Phagedœnic* or *sloughing*, rapid progress, eroding action, and no induration at the base. *Syphilitic*, indurated base, elongated, or irregular in shape, and does not rapidly extend. *Cancerous* ulcers are, circular shape, "eroded edges," and spread rapidly.

These are the main characteristics. The microscope should not be neglected, however, in examining sores in this region supposed to be cancerous. The cancerous ulcers produce all the dreaded symptoms and accompaniment of cancers elsewhere, and are very fatal. Joined to the proper cancerous disease is the inability to eat, which renders the condition of the patient more deplorable.

*Abscess of the Gums* is caused by the roots of decayed teeth, cold, or some other inflammation, and sometimes results in the formation of pus. It is of trifling moment, although it gives rise in many instances to considerable suffering. Remedies act very well. Evacuate the pus.

*Ulceration of the Gums.*—Syphilis, scrofula, cancer, or any of the conditions operating to produce similar lesions in the mouth and on the tongue, may have the same effect here.

*Epulis.*—This is a tumor of a fibrous character springing from the periosteum and edge of the alveolus, and implicating the osseous walls, growing up between and loosening the neighboring teeth, which it displaces and envelopes in its structure. It is most frequently met with in the lower jaw, and commonly about the molar teeth. This tumor is red, smooth, and lobulated, at first hard and semi elastic, like the ordinary structure of the gum, but after a time softening by disintegration, and ulceration on the surface, with a purulent or sanious discharge ; it appears to be simply circumscribed hypertrophy of the gum.

*Malignant Epulis* is soft, purplish, very vascular, grows rapidly, and is specially produced after removal. They are principally found in men advanced in life.—*Medical Investigator.*

